# Elucidating Decorin’s role in the preovulatory follicle

**DOI:** 10.1186/s13048-020-0612-3

**Published:** 2020-02-10

**Authors:** A. Kedem, K. Ulanenko-Shenkar, Y. Yung, G. M. Yerushalmi, E. Maman, A. Hourvitz

**Affiliations:** 1 Human Reproduction Lab and IVF Unit, Department of Obstetrics and Gynecology, Chaim Sheba Medical Center, Affiliated to Tel Aviv University, Sackler Faculty of Medicine, Tel-Hashomer, Ramat Gan, Israel; 2 IVF unit, Shamir Medical center (Assaf Hrofeh), Affiliated to Tel Aviv University, Sackler Faculty of Medicine, Tel-Aviv, Israel

**Keywords:** *DCN*, Granulosa cells (mural and cumulus), Ovulation, IVF, Preovulatory follicles, Migration, ECM

## Abstract

**Background:**

*DCN* (decorin) is a proteoglycan known to be involved in regulating cell proliferation, collagen fibril organization and migration. In our global transcriptome RNA-sequencing approach to systematically identify new ovulation-associated genes, *DCN* was identified as one of the highly regulated genes. We therefore hypothesize that DCN may have a role in ovulatory processes such as cell migration and proliferation.

**Aim:**

To characterize the expression, regulation and function of the proteoglycan *DCN* in the human ovarian follicles during the preovulatory period.

**Methods:**

The in-vivo expression of *DCN* mRNA in mural (MGCs) and cumulus (CGCs) granulosa cells was characterized using quantitative RT-PCR and western blot.

A signaling study was performed by treating human MGCs cultures with gonadotropins and different stimulators and inhibitors to determine their effect on *DCN* expression by qRT- PCR and elucidate the pathways regulating these proteins.

In a functional study, KGN granulosa cell line was used to study cell migration with a scratch assay.

**Results:**

*DCN* mRNA expression was significantly higher in MGCs compared to CGCs. *DCN* mRNA was significantly higher in CGCs surrounding mature metaphase II (MII) oocytes compared to CGCs of germinal vesicle (GV) and metaphase I (MI) oocytes.

hCG significantly increased *DCN* mRNA and protein expression levels in cultured MGCs. Using signal transduction activators and inhibitors, we demonstrated that *DCN* induction by LH/hCG is carried out via PKA, PKC, ERK/MEK, and PI3K pathways.

We showed that *DCN* expression is also induced in high-density cell cultures, in a dose-dependent pattern. In addition, progesterone induced a significant increase in *DCN* secretion to the media. MGCs from follicles of endometriosis patients exhibited reduced (about 20% of) mRNA transcriptions levels compared to MGCs follicles of control patients. More significantly, we found that *DCN* has an inhibiting effect on KGN cell migration.

**Conclusions:**

Our study indicates that *DCN* is a unique ovulatory gene.

Our findings support the hypothesis that *DCN* plays an important new role during the preovulatory period and ovulation, and stress its involvement in endometriosis infertility. A better understanding of *DCN* role in ovulation and endometriosis may provide treatment for some types of infertility.

## Introduction

Ovarian follicular development and ovulation in humans are highly complex and tightly regulated events [1–3]. In order to characterize the final stages of follicular maturation and ovulation pathways, a transcriptome sequencing was used in our laboratory to identify differences in gene expression between the cumulus of a compact cumulus-oocyte complex (COC) with a GV stage oocyte, and an expanded COC with an MII stage oocyte [4]. Based on that study, the proteoglycan *DCN* was among the most upregulated genes in our ovulatory gene library.

*DCN* is a member of the small leucine-rich proteoglycan (SLRP) family. It is synthesized mainly by fibroblasts, stressed vascular endothelial cells and smooth muscle cells. *DCN* is associated with the extracellular matrix (ECM) and has a high-affinity interaction with collagen fibers and subsequently regulates collagen fibrillogenesis.

*DCN* is remarkable in that it can interfere with the signaling of growth factors (GFs [5];). This may be relevant to the regulation of the immature primate testis and in states of human male infertility. In both of these situations, *DCN* is massively increased and may interfere with paracrine signaling of GFs [6, 7].

These interactions are consistent with *DCN’s* involvement in diverse pathological processes such as tumor growth and metastasis [8], angiogenesis [9], renal and pulmonary fibrosis [10], muscular dystrophy [11], wound healing and myocardial infarctions [9, 12].

A few studies have examined *DCN’s* role in the human and primate ovarian extracellular. Using immunohistochemistry in adult rhesus monkey and human ovaries, it was found that *DCN* is expressed in theca cells, the corpus luteum (CL), and follicular fluid (FF) [13] Moreover, it was shown that adding *DCN* to cultured CGs, elevated intracellular Ca2+ levels and phosphorylation of EGFR. Based on these results the study suggested that *DCN* plays multiple roles in granulosa cells, including folliculogenesis, ovulation, and survival of the CL [14]. More recent work demonstrated that *DCN* regulates apoptosis and cell cycle of granulosa cells in goat ovaries [15]. *DCN* has been previously biochemically identified in the extract of bovine ovarian follicle; it appeared to be localized throughout the ovary wherever structural collagen exists [16]. Finally, SLRP family members like *DCN* have been shown to bind and modify the assembly kinetics of fibrillar collagen [17], and are both substrates of ADAMTS enzymes that have an important role in regulating the ovulation process [18].

Based on these data, we hypothesized that *DCN* is a unique ovulatory gene. To study the role of DCN in the follicle we established an in vivo and in vitro approach to characterize the expression, regulation and DCN function in the human ovarian follicles during pre- and post-ovulatory period.

## Materials and methods

### Study design and patients

A prospective study involving 49 patients treated with ART was performed at Chaim Sheba Medical Center, Tel Hashomer. This study has 3 parts:

1. In-vivo expression of DCN mRNA: In order to characterize *DCN* mRNA expression in relation to follicular size and oocyte maturation, mural (MGCs) and cumulus (CGCs) granulosa cells were collected from patients undergoing IVF or IVM procedures and were analyzed using quantitative RT-PCR and western blot.
2. Signaling study of the pathways regulating DCN: Human MGCs were collected during IVF procedures and cultured. These cells were treated with gonadotropins and different stimulators and inhibitors to determine their effect on *DCN* expression by qRT- PCR.
3. Functional studies were performed to identify the role of decorin in the ovulatory process:
  - KGN – granulosa cell line was used for a cell migration study with a scratch assay.
  - The effect of DCN on proliferation of MGCs and KGN granulosa cell lines was examined.
  - The effect of progesterone on DCN secretion in MGCs was studied.
  - The role of DCN in endometriosis.

The study was approved by the local Institutional Review Board (IRB) committee of Chaim Sheba Medical Center, Tel Hashomer (ethical approval number SMC-17-4521). Written informed consent was obtained from each patient who provided samples. All experiments involving mice were conducted in compliance with the principles of the National Research Council (NRC) and were approved by the institutional animal care and use committee (IACUC) #919/14/ANIM.

### In vitro fertilization (IVF) protocol

MGCs were obtained from large follicles at the time of oocyte retrieval from women.

< 40 years of age undergoing IVF treatment due to mechanical problems, endometriosis (confirmed by surgery or ultrasound), pre-implantation genetic disorders, or male factors. Stimulation protocols were used for the induction of follicular growth as previously described [19]. All patients underwent suppression using a GnRH antagonist protocol (0.25 mg/day, Cetrorelix, Cetrotide; Merck-Serono, Darmstadt, Germany). Ovarian stimulation was performed with a daily subcutaneous dose of recombinant FSH (either Gonal-F; Merck Serono, Darmstadt, Germany,or Puregon Pen,Schering Plough, North Wales PA, USA), which was commenced on the third day of the menstrual cycle and was continued for 5 days. This was followed by a daily dose of human menopausal gonadotrophin (Menogon, Ferring, Switzerland). The initial dose used was dependent upon age, body mass index and prior IVF treatment history. When 3 leading follicles had reached 18 mm in diameter, patients received 250 μg human chorionic gonadotrophin (hCG) (Ovitrelle, Merck-Serono, Darmstadt, Germany).

Oocyte retrieval was scheduled for 36 h after hCG injection and performed by transvaginal ultrasound-guided needle aspiration. FFs were collected in culture tubes containing flushing medium (MediCult).

### In vitro maturation (IVM) protocol

In the hormonal-stimulated protocol, normo-ovulatory patients underwent an in vitro maturation (IVM) cycle according to accepted IVM protocols [20]. Accordingly, a baseline evaluation that included a hormonal profile and an ultrasound scan was performed on days 2–3 of the menstrual cycle. On day 3, a 150 IU/day recombinant FSH (GONAL-f, Merck-Serono, Darmstadt, Germany) was added for 3 days. A second evaluation was performed on day 6 of the menstrual cycle. An injection of 10,000 IU hCG (Pregnyl; Merck, NJ, USA) was administered subcutaneously when the endometrial thickness was 5 mm and the leading follicle was at least 12 mm. Transvaginal oocyte retrieval was scheduled for 36–38 h after hCG injection. During oocyte aspiration, the follicles were measured, and the FF was separated into 2 groups of small (< 10 mm) and large follicles (> 10 mm).

### Mural granulosa cell collection and grouping

MGCs were collected from the FF and were re-suspended in a phosphate-buffered solution (PBS, Sigma St Louis MO, USA). After allowing the cells to settle by gravity for a few minutes, the top medium was aspirated. This step was repeated 2–3 times until the medium became clear. MGCs were enriched by centrifugation at 1800 rpm for 15 min using the 40/80 Ficoll-Hypaque gradient (Sigma Aldrich St Louis MO). The enriched MGCs were collected into a new tube, were washed with PBS to remove residues of the gradient and were centrifuged again at 1000 rpm for 5 min. Purified MGCs of 3–4 women undergoing IVF procedures were pooled and represented a biological replicate. Pooled cells were subjected to total RNA purification.

Purified MGCs (< 14 mm) from follicles of women undergoing IVM procedures were grouped, each group containing MGCs from three to four different women to represent a biological replicate. Pooled cells were subjected to total RNA purification.

### Cumulus cell collection and grouping

CGCs were obtained during oocyte denudation for the intracytoplasmic sperm injection (ICSI) procedure. After oocyte retrieval, CGCs of each oocyte were removed by hyaluronidase (SAGE, Trumbull, CT, USA) and a glass denudation pipette (Swemed, Billdal, Sweden). The cells were washed in PBS and centrifuged at 5000×*g* for 5 min at room temperature. The resulting pellets were stored at − 80 °C until RNA isolation.

Cumulus cells isolated from individual oocytes were divided into different groups according to their corresponding oocyte maturation stage: CGC_GV_, CGC_MI_, CGC_MII_. Each group contained CGCs derived from 3 to 4 different women and represented a biological replicate.

### Mural granulosa cell culture

Follicular fluids, containing MGCs, were collected during IVF procedures. Cells were aspired from follicular fluids and washed with PBS until the medium became clear.MGCs were enriched by centrifugation at 1800 rpm for 15 min using 40/80 Ficoll-Hypaque gradient (Sigma Aldrich St Louis MO). The enriched MGCs were collected into a new tube, residues of gradient were washed away with PBS and the cells were centrifuged again at 1000 rpm for 5 min. The resulting pellets were re-suspended in basic medium (Medium 199, Sigma Aldrich St Louis Mo) supplemented with 5% FCS (Invirogen Grand Island, NY) and 1% penicillin/streptomycin (Sigma Aldrich, St Louis, Mo). MGCs were plated in 24-well plates at a density of 100,000 cells/well and incubated at 37 °C in a humidified atmosphere with 5% CO2 in air.

In order to create an hCG (Ovitrelle, Serono, Italy) response timeline, MGCs were cultured as described in a basic medium with a daily medium exchange. Each day (1 through 6) MGCs were supplemented with either 1 U/ml or 10 U/ml hCG. A control group of MCGs had no hCG added. At different times, as indicated, cells were harvested and frozen for further analysis. In order to study signaling pathways regulating *DCN,* MGCs were stimulated with 1 or 10 U hCG, 10 μM FSK, 20 nM PMA or 100/300 nM Progesterone (Dienogest pill dissolved in DMSO) and pretreated with specific inhibitors, 10 μM LY 294002, 10 μM H89, 10 μM U0126, (all from LC Laboratories, Woburn, MA, USA). MGCs were harvested 24 h after stimulation.

### RNA extraction and qRT-PCR

Total RNA was extracted from MGCs or CGCs using a Mini/Micro RNA Isolation kit (Zymo Research, CA, USA), according to the manufacturer’s instructions. RNA purity and concentration were assessed using nanodrop (NanoDrop 2000C, Thermo Scientific Waltham, MA). Of the total RNA from each sample, 25 μg was used for cDNA synthesis using a High Capacity Reverse Transcription kit (Applied Biosystems, Carlsbad, CA) according to the manufacturer’s instructions. mRNA levels were analyzed by real-time PCR using StepOnePlus real-time PCR system (Applied Biosystems. Carlsbad, CA). Real-time PCR mix contained 1 μl of cDNA, fast SYBR Green Master Mix (Applied Biosystems) and specific primers for *DCN* and β-actin (housekeeping gene) in a total volume of 10 μl. All samples were subjected to qRT-PCR in triplicates. Analysis of qRT-PCR results was carried out using StepOne software. Relative gene expression was calculated using the delta-delta Ct method. Details of the primers used are shown in Table 1.

**Table 1:** List of primers sequences used in the study

Gene	Primer sequence	Product size	Accession Number
Actin_F	TTGCCGACAGGATGCAGAA	78 bp	Diabetes 60:936–946, 2011
Actin_R	GCTCAGGAGGAGCAATGATCTT		
Decorin_F	GGAATTGAAAATGGGGCTTT	221 bp	NM_001920
Decorin_R	GCCATTGTCAACAGCAGAGA		

### Western blot

Cells were harvested using 0.5 mL PBS and pelleted. Cell pellets were lysed in TNE buffer (50 mM Tris–HCl, pH 8.0, 250 mM NaCl, 2 mM EDTA, 1% NP-40, Sigma Aldrich St Louis, MO, USA) and a protease inhibitor cocktail (Sigma–Aldrich, St. Louis, MO, USA), vortexed and incubated for 10 min on ice before removal of nuclei and debris by centrifugation. Aliquots of the clarified supernatants were used to determine protein concentration. Protein concentration was determined by the Bradford method (Protein Assay Dye Reagent, Bio-Rad, Hercules, CA, USA). Equal amounts (50 μg) of protein were loaded and separated on SDS-Polyacrylamide gel (10% acrylamide). Proteins were then transferred onto nitrocellulose membranes. Membranes were blocked in 5% bovine serum albumin (BSA) in TBST (100 mL TBS 10X, 900 mL H_2_O, 1 mL Tween 20, Sigma Aldrich St. Louis, MO, USA) for 1 hour and afterwards incubated with a primary antibody against *DCN* (R&D systems, USA, 1 μg/ml) or β-actin (1:1000 SantaCruzBio Dallas, Texas USA housekeeping gene) overnight at 4 °C. The membranes were then treated with a horseradish peroxidase-conjugated secondary antibody and developed using an enhanced chemiluminescence kit (Sigma Aldrich St Louis, MO, USA).

### ELISA for *DCN* levels in cell culture media

MGCs were seeded at a density of 1 × 10^5^ cells per well in 24-well plates and cultured for 4 days with daily media exchange before being treated with 1 unit/ml of hCG. The media were then collected and the level of *DCN* was assayed using the *DCN* Human ELISA kit (BOSTER, CA, sensitivity - < 10 pg/ml) according to the manufacturer’s instructions.

### PCNA staining

MGCs or KGN cells were seeded at a density of 0.8 × 105 cells per well in 12-well plates. MGCs were cultured for 48 h before adding 5-20 μg/ml of human recombinant DCN for an additional 24 h incubation. For KGN cells, human recombinant DCN was added 24 h after seeding, for an additional 24 h. Fixation was performed with 4% PFA for 20 min. at room temperature followed by washing 3 times with PBS. Fixated cells were treated with Peroxidase block containing 90% Methanol, 2% H2O2 (30%) and 8% DDW for 10 min at room temperature and washed 2 times with PBS. CAS-block (Invitrogen, USA) was added for 10 min. Anti-PCNA antibody (1:100,Dako, Denmark) was added for 1 h at room temperature. Cells were washed 3 times with PBS before a secondary antibody (1:100) was added for 30 min, followed by × 3 PBS wash. Finally, DAB Substrate (Thermo scientific, USA) was used according to manufacturer’s instructions. Cells were stained using hematoxylin (Sigma St Louis MO, USA), and pictures were taken straightway.

### Scratch assay

KGN cells were seeded in 24-well plates and once 80% confluence was reached, a vertical midline scratch was performed using a 10 μl tip with a blunt surface. Photographs were then taken with a Nikon digital sight camera attached to a Nikon Eclipse TS100 microscope at 10× magnification. Immediately thereafter, the medium was changed to starvation medium and 40 μg/mL DCN (R&D Systems, Minneapolis, MN) was applied for 24 h. Photographs were again taken at 18 and 24-h point and were then analyzed using ImageJ® Imaging Software (NIH, USA). Experiments were run in triplicate and wound distances were compared before and after treatment, within and between treatment groups.

### Statistical analysis

Data was expressed as mean ± SEM and evaluated with Student’s T-test (two-tailed) or with ANOVA for more than two variances, using the post hoc Tukey test assuming equal variances or the Games-Howell test for unequal variances. For all statistical analysis, SPSS 19 software (IBM, Armonk NY) was used. *P* values < 0.05 were considered statistically significant.

## Results

### DCN expression in vivo

#### *DCN* mRNA expression is higher in MGCs compared to CGCs

**Fig. 1:**
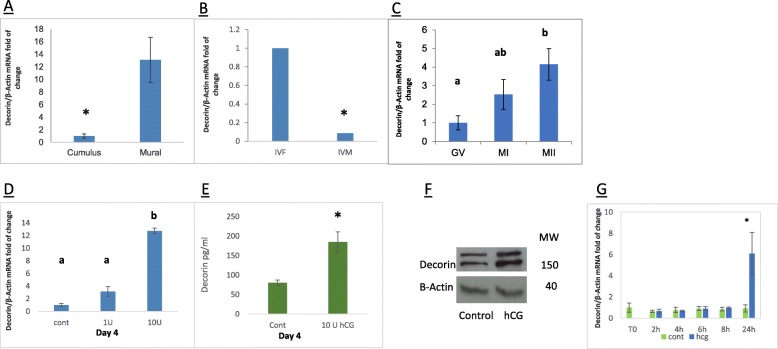
**a**. Expression of *DCN* in CGCs and MGCs. CGCs and MGCs were aspirated from preovulatory follicles (> 17 mm) of woman undergoing IVF and purified. Cells from 3 to 4 women were grouped and total mRNA was subjected to qPCR with DCN and b-actin primers. *DCN* was quantified by qPCR and normalized to *β-actin* expression. Data represent the mean ± SEM of three independent experiments. Gene expression was calculated as fold change relative to CGC that was set to 1. (*P* = 0.02). **b**. *DCN* mRNA expression in MGCs obtained from different stages of follicular development. MGCs were collected during IVM from small antral follicles (< 14 mm) and during IVF procedures from preovulatory follicles (> 17 mm). Data represent the mean ± SEM of three independent experiments. Gene expression was calculated as fold change relative to IVM that was set to 1. (*P* = 0.001). **c**. *DCN* mRNA expression in CGCs according to maturation stage of the related oocyte. CGCs were collected from cumulus-oocyte complex retrieved from women undergoing in-vitro fertilization (IVF) procedure. Cells were grouped according to the maturation stage of the related oocyte: GV, MI, and MII. Extracted mRNA was subjected to qPCR with *DCN* primers and b-actin primers. Data represent the mean ± SEM of three independent experiments. Gene expression was calculated as fold change relative to GV that was set to 1. (*P < 0.05)*. **d**. Characterization of the optimal incubation time for hCG effect on *DCN* mRNA expression in MGCs cell culture. MGCs were collected during IVF procedure and cultured. hCG (1 or 10 unit/ml) was added to the medium after 0–6 incubation days with a daily medium exchange. Cells were collected 24 h after hCG stimulation and the total mRNA was subjected to qPCR with *DCN* primer and b-actin primers. Data represent the mean ± SEM of three independent experiments. Gene expression was calculated as fold change relative to the control sample that was set to 1. Different letters represent significant results. (*P < 0.05)* (**e**) The conditioned media of the experiment in **d** was collected for DCN concentration measurement using EIA kit. Concentrations are in absolute values. * represent significant differences (*P* < 0.05). The results are expressed as mean ± SEM of three independent experiments. **f** The protein level of DCN was determined by western blotting in three independent experiments. Actin was used as a control. **g***DCN* mRNA expression in MGCs cell culture during 24 h. MGCs were aspirated from preovulatory follicles (> 17 mm) during IVF and cultured for 4 days with a daily medium exchange, then, 10 unit/ml of hCG was added to the medium. Cells were collected at starting point and at 2 h, 4 h, 6 h, 8 h, and 24 h. Data represent the mean ± SEM of three independent experiments. Gene expression was calculated as fold change relative to T0 sample that was set to 1. (P DCN = 0.003). * marks the significant result

In order to characterize the expression of *DCN* in the follicle, MGCs and CGCs from large preovulatory follicles (> 17 mm) were collected during IVF procedures. The results showed that MGCs exhibited significantly higher *DCN* mRNA levels compared to CGCs (Fig. 1a).

#### *DCN* mRNA expression levels increase during final follicular growth

To expand our knowledge regarding the mRNA expression in vivo*,* we evaluated the *DCN* mRNA expression pattern in MGCs during late antral follicle development. Gene expression was assessed in MGCs obtained from two different sized follicles: small (< 14 mm) follicles obtained from IVM procedures and preovulatory (≥17 mm) follicles obtained from women undergoing IVF procedure. Results revealed that MGCs of preovulatory follicles exhibit significantly higher levels of *DCN* mRNA expression (Fig. 1b).

#### *DCN* mRNA levels are higher in CGCs surrounding a mature oocyte

To determine whether the expression of *DCN* transcripts is related to oocyte maturation status, we used CGCs denuded from GV, MI, or MII COCs aspirated from women undergoing IVF. As shown (Fig. 1c), the expression of *DCN* transcripts is significantly higher in CGCs from MII COCs compared to CGCs from GV and MI COCs.

### *DCN* expression in cultured human mural granulosa cells

#### hCG effect on *DCN* mRNA

To understand more about the role of *DCN* during ovulation we explored the effect of hCG on *DCN* mRNA expression, in vitro, using MGC cell cultures. As shown previously, [21, 22] MGCs in culture regain hormonal responsiveness after incubation for 4 days with a daily medium exchange. First, MGCs were collected and treated as described in Materials and Methods, with a daily medium exchange. Cultured cells were treated with 0, 1 or 10 unit/ml of hCG at various days after incubation. Twenty-four hours after stimulation, cells were harvested for mRNA and protein studies, while media were collected to measure the levels of secreted DCN protein. qPCR results revealed that hCG treatment elevates mRNA expression of *DCN* significantly after 3, 4 and 5 days of culture (Fig. 1d). The most significant effect was detected in cells treated with 10 U/ml hCG after 4 days of culture (about 13-fold). At day 4, the induction of the DCN protein by hCG is shown in the cell extract as well as in the medium (Fig. 1e-f). Taken together, results indicate that the *DCN* gene is induced by hCG and that the highest level of induction is achieved after 4 days of culture.

To determine whether *DCN* is an early or late hCG response gene, MGCs were incubated for 4 days with a daily medium exchange. Following the first stage, 10 unit/ml of hCG was added and cells were collected at baseline, as well as at 2 h, 4 h, 6 h, 8 h, and 24 h. Results show that the hCG trigger elevated *DCN* mRNA expression only 24 h after treatment, suggesting that *DCN* is a late response target of hCG (Fig. 1g).

#### Elucidation of the intracellular signaling pathways mediating hCG induced *DCN* expression

It has been previously shown that LH/hCG activates both protein kinase A (PKA) and protein kinase C (PKC) signaling pathways to induce the expression of preovulatory genes [23–25]. In addition, phosphoinositide 3-kinase (PI3K)/Protein kinase B (Akt) has been reported to be involved in LH receptor downstream signaling [26, 27].

To investigate whether PKA, PKC and/or PI3K signaling pathways are involved in the hCG-induced upregulation of *DCN* mRNA, MGCs were treated with either 20 nM phorbol myristate acetate (PMA) or 10 μM forskolin (FSK) respectively, to activate the PKC and PKA pathways.

**Fig. 2:**
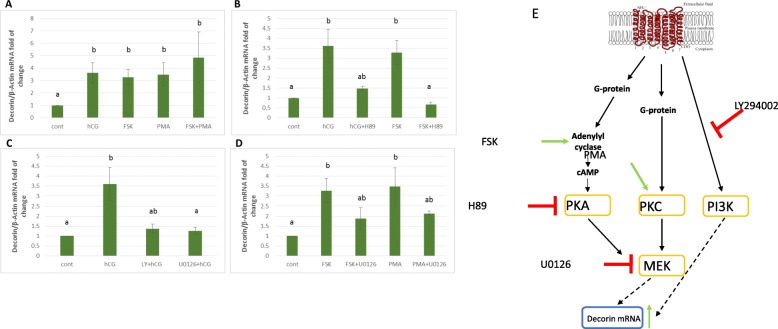
Involvement of PKA, PKC, MEK/ERK and PI3K pathways in LH-induced expression of *DCN*. MGCs collected during IVF procedures were cultured for 4 days with daily medium exchanges. Then, MGCs were either stimulated with (**a**) vehicle (control), hCG 10 U/ml, 10 μM forskolin (FSK), 20 nM PMA, or with a combination of 10 μM FSK and 20 nM PMA for 24 h. Cells were pretreated for 1 h with H-89 (10 μM), U0126 (10 μM), or LY 294002 (10 μM), and then stimulated with 10 U/ml hCG, 10 μM FSK, or 20 nM PMA for 24 h. Total mRNA was subjected to qPCR with *DCN* (**a**-**d**) primers and b-actin (housekeeping gene) primers. *DCN* was quantified by qPCR and normalized to *β-actin* expression. Data represent the mean ± SEM of three independent experiments. Different letters represent significant differences (*p* < 0.05). **e** Schematic representation of LH/hCG signaling cascade that mediates *DCN* expression in MGCs

As shown in Fig. 2a FSK, an activator of adenylate cyclase (AC), elevated the expression of DCN at levels similar to that of hCG (3.5 fold induction), while the PKC activator, PMA, stimulated the expression of *DCN* expression in a similar manner. Treating MGCs with PMA and FSK simultaneously led to 4.5-fold induction of *DCN* mRNA (Fig. 2a).

To further confirm the role of PKA in hCG-induced upregulation of *DCN*, the selective PKA inhibitor, H89, was used to block PKA function. MGCs were pre-treated with 10 μM H89 for 1 h and subsequently treated with either 10 U hCG or 10 μM FSK for 24 h. qPCR results show that hCG- and FSK-induced upregulation of *DCN* mRNA levels were blocked by pretreatment with H89 (Fig. 2b).

To examine the involvement of PI3K signaling in the hCG- induced *DCN* up-regulation, MGCs were treated with 10 U hCG for 24 h with or without 1 h pre-treatment with 10 μM LY294002 (a PI3K inhibitor). RT-qPCR results revealed that hCG-induced up-regulation of *DCN* mRNA levels were blocked by pre-treatment with LY294002 (Fig. 2c).

Taken together, these results indicate that hCG-induced upregulation of *DCN* is regulated by PKA, PKC and PI3K pathways.

The extracellular signal-regulated kinase (ERK) has been reported to be involved in LH receptor downstream signaling [26, 27]. Based on this information, we evaluated roles for ERK pathways in hCG-induced *DCN* upregulation.

To examine the involvement of ERK signaling in the hCG- induced *DCN* up-regulation, MGCs were treated with 10 U hCG for 24 h with or without 1 h pre-treatment with 10 μM U0126 (an inhibitor of mitogen-activated protein kinase (MEK), an upstream activator of ERK). Pre-treatment of MGCs with U0126 completely abolished hCG-induced up-regulation of *DCN* mRNA (Fig. 2c). These results suggest that the ERK/MEK pathway is involved in the signaling pathway evoked by activation of LHR to promote *DCN* up-regulation, together with PI3K/Akt pathway which also participates in this signaling cascade. We also show that blocking ERK/MEK using U0126 inhibits the DCN up-regulation by PKA and PKC pathways (Fig. 2d).

#### Assessment of the effect of MGCs confluence on *DCN* expression

**Fig. 3:**
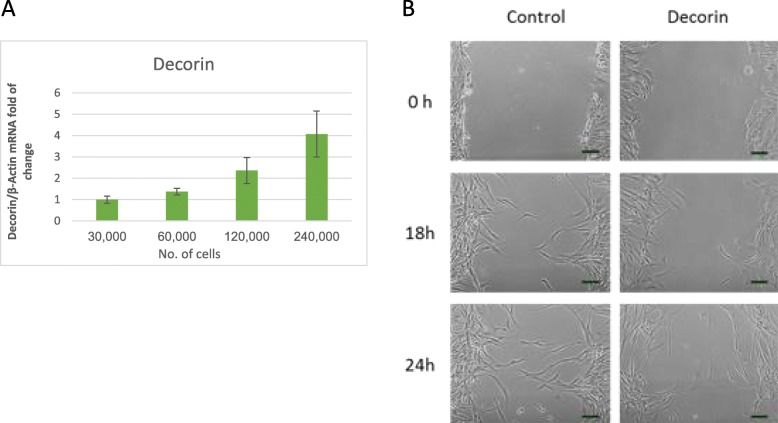
**a**. *DCN* mRNA expression in MGCs cultures seeded in different concentration. MGCs collected during IVF procedure and cultured in different concentrations as indicated. After 48 h, cells were collected and Total mRNA was subjected to qPCR with *DCN* and b-actin (housekeeping gene) primers. Data represent the mean ± SEM of four independent experiments. Gene expression was calculated as fold change relative to 30,000 cell sample that was set to 1. Different letters represent a significant difference (*P < 0.05).***b** The effect of *DCN* on KGN cell migration. Representative. Cells were cultured to full confluence and a scratch was inflicted with and without the addition of DCN. The cells were then cultured in starvation medium for additional 24 h. Images were taken at 0 h, 18 h, and 24 h. The results show representatives of time-lapse images of migrating KGN cells out of three independent experiments. Scale bar = 100 μM

Previous works showed that *DCN* is involved in proliferation inhibition [28–31]. Accordingly, we hypothesized that *DCN* may have a role in inhibiting cell proliferation in the follicle. It is a known phenomenon that cell confluence inhibits proliferation of non-transformed cells via cell to cell contact [32]. To examine the expression of *DCN* at different confluence levels, MGCs were collected from women undergoing IVF procedures, and cultured in different densities. Total mRNA was subjected to qPCR. Results show that *DCN* levels are significantly higher in high-density cell cultures, in a dose-dependent manner (Fig. 3a).

#### Exogenous DCN does not inhibit MGCs or KGN proliferation in vitro

Based on results exhibited in Fig. 3a, we wanted to determine the involvement of DCN in MGCs and KGN proliferation regulation. We chose to examine the effect of DCN on MGCs and KGN proliferation using the PCNA method. MGCs were collected from women undergoing IVF, and cultured. Medium was changed daily and various concentrations of DCN were added. Cells were cultured for 72 h, fixed and stained for PCNA proliferation assay. KGN cells were cultured for 24 h and treated the same way. In each sample two areas were examined, a dense high confluence area, and, a low confluence area with individual cells. Both DCN treated cells and control groups showed a similar proliferation pattern in which low confluence cells underwent proliferation, unlike cells in denser areas that did not proliferate, probably due to contact inhibition. Both DCN treated cells and control groups showed a similar proliferation pattern at all DCN concentrations tested (Additional file 1: Figure S1A and Figure S1B). Both, KGN and MGCs results support the notion that DCN does not inhibit cell proliferation in vitro*.*

#### The effect of DCN on KGN migration

As described previously [28, 33–36], DCN was found to be involved in inhibition of cell migration. In order to test DCN effect on migration of KGN cells, the cells were seeded and cultured for 24 h before the surface of the well was scratched to create a gap between cells (scratch test). The medium was changed to starvation medium in order to prevent cell proliferation, and DCN was added to the medium. Images were taken at 0 h, 18 h, and 24 h (Fig. 3b). We found that DCN has an inhibiting effect on KGN cell migration.

#### The effect of progesterone on *DCN* expression

**Fig. 4:**
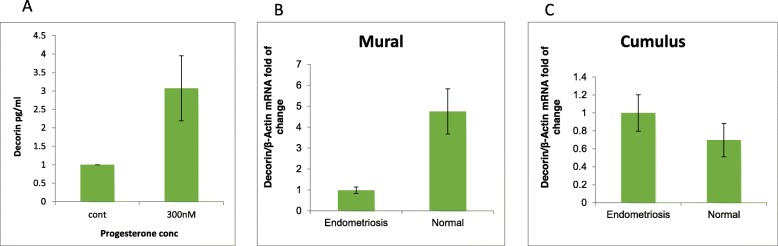
**a:** The effect of progesterone on DCN secretion. MGCs were aspirated from preovulatory follicles (> 17 mm) during IVF and cultured for 4 days with a daily medium exchange. 300 nM of progesterone was added to the media for 24 h. Media was collected and subjected to EIA kit measuring *DCN* concentration. Concentrations are in absolute values. The results are expressed as mean with ± SEM of 4 independent experiments. * represents significant result (*P*- 0.02). **b-c**: Expression levels of decorin in CGCs and MGCs obtain from follicles of endometriosis patients. CGCs and MGCs were aspirated from preovulatory follicles (> 17 mm) of endometriosis patients and from control patients. Cells of 3–4 patients were grouped, total mRNA was extracted and subjected to qPCR with decorin and b-actin primers. The results are expressed as fold change mean ± SEM of three independent experiments. Endometriosis sample was set to 1. (P decorin = 0.008). * marks significant result

A recent study [37] found that *DCN* expression is induced by progesterone in endometrium immortalized cell lines taken from ovarian endometriomas. In order to evaluate a possible physiological post-ovulation role of DCN we examined the effect of progesterone on DCN secretion in MGCs culture. Cells were collected during IVF procedures and cultured for 4 days with a daily medium exchange. Then, cells were treated with 300 nM of progesterone for 24 h. Medium from cells was collected and subjected to EIA kit measuring DCN concentration. Results indicate that 300 nM of progesterone induces a significant increase (*p* = 0.02) in DCN secretion to media compared to untreated cells (Fig. 4a). Based on results exhibited in Fig. 4a and in previous publications [28, 29, 31, 36, 38–40], we wanted to determine the involvement of *DCN* in MGCs and KGN proliferation regulation using the PCNA method [41] (see Materials and Methods). DCN treated cells and control groups showed a similar proliferation pattern in all DCN concentrations tested. The results (Additional file 1) show that DCN did not inhibit in vitro cell proliferation of KGN or of MGCs.

#### DCN expression in MGCs obtained from endometriosis patients

It has been previously shown that induction of *DCN* by progesterone plays a crucial role in suppressing endometriosis [37]. However, it was not clear whether DCN was involved in the pathology of endometriosis. In order to elucidate the role of DCN in endometriosis and to examine whether DCN expression is dysregulated in MGCs and CGCs of women with endometriosis, cells from large preovulatory follicles (> 17 mm) were collected during IVF procedures. Granulosa cells from patients that were previously diagnosed with severe endometriosis were compared to control patients with no endometriosis. Samples from 3 to 4 women were grouped and total mRNA was subjected to qPCR to determine mRNA levels of *DCN* in both cell types. Results show that *DCN* expression in MGCs from follicles of endometriosis patients exhibit a 5-fold reduction compared to MGCs follicles of control patients (Fig. 4b, *p* = 0.008). DCN levels in CGCs were not significantly different in endometriosis patients (Fig. 4c).

## Discussion

Ovarian follicular development and ovulation in humans are complex events that are controlled and regulated by a large number of genes. Identifying new genes involved in these processes is of major significance to research and to clinical application. We recently applied the RNA sequencing (RNASeq) method in order to systematically isolate genes with an ovulation-selective pattern of expression [4]. *DCN* was found to be a highly up-regulated gene and therefore it was selected for further investigation of its ovulation-associated expression patterns and function.

In a recent study, Sawada et al. showed high FF concentration of *DCN* in mature follicles: however, *DCN* was not detected in GCs of mature follicles using immunohistochemistry and western blot and the source of DCN was unclear [42]. In the present study we revealed a clear expression and secretion of *DCN* in MGCs and cumulus GCs obtained from mature and immature follicles (Fig. 1). We have been able to demonstrate that *DCN* mRNA is almost exclusively expressed in hCG-triggered preovulatory follicles (IVF) and minimally in hCG-triggered small antral follicles (IVM). In addition, we demonstrated that DCN mRNA levels are significantly higher in MGCs compared to CGCs (Fig. 1a). These findings concur with higher LHCGR expression in mature follicles compared to immature follicles and in MGC compared to CGC. As a result, hCG-induced expression of DCN is higher in preovulatory follicles [43–45].

In this study, in order to identify the optimal time for HCG stimulation, we designed a time point experiment. We concluded that applying HCG after 4 days of cell culture incubation yields an optimal response. Furthermore, we have previously shown [22] that some genes exhibit a fast response to hCG while others are induced at a later time point. Here we have demonstrated that DCN is a late LH/hCG induced gene (Fig. 2).

Several researchers have reported that CGCs’ gene expression patterns could be used as a noninvasive tool to predict oocyte competence or embryo development [46, 47].

We found that *DCN* expression in human CGCs is correlated with the maturation stage of their related oocyte. Expression was lower in cumulus cells obtained from GV and MI oocytes compared to MII oocytes (Fig. 1c). One possible explanation for this expression pattern is that oocytes that failed to undergo maturation under hyper-stimulatory conditions were unable to do so and as a consequence, their cumulus cells failed to express *DCN*. Another assumption is that the reduced expression of *DCN* in CGCs is one of the causative factors for delayed oocyte maturation. These assumptions need to be further investigated and verified.

Little is known about the signaling pathways mediating the up-regulation of *DCN* in MGCs. Using an in vitro experimental model [32], we further investigated the signaling pathway by which ovulatory hCG induces *DCN* expression in preovulatory. MGCs.

LH receptor activation leads to the activation of adenylate cyclase (AC) and to the production of the secondary messenger, cAMP [48]. The LH signal further activates a myriad of intracellular signaling pathways, including PKA, PKC, PI3K, and ERK1/2 [23, 24, 26, 27, 49]. We found that both the PKA and PKC pathways appear to be involved in the regulation of *DCN* expression since either FSK or PMA were observed to induce its up-regulation. Co-treatment with to FSK and PMA lead to increased induction of *DCN* (Fig. 2a). It may thus be concluded that PKA and PKC signaling systems are co-interacting to induce *DCN* mRNA expression. Indeed, accumulating *evidence suggests* that activation of a single signaling cascade is not sufficient to activate target gene expression, and that cross-talk between and among signaling cascades are required [50]. Previous studies have reported that PKA and PKC can activate ERK1/2 in MGCs [42, 49]. In the present study, treatment of MGCs with the MEK inhibitor abolished the FSK and PMA effects on *DCN* mRNA expression thereby indicating that MEK is a downstream component of PKA and PKC pathways (Fig. 2d). Herein, we also describe for the first time the intracellular signaling pathways that regulate *DCN* mRNA expression in GCs. As depicted in Fig. 2e, the hCG-dependent induction of *DCN* expression in preovulatory MGCs is mediated by the cAMP-PKA and PKC pathways which exert their effects by activating the MEK/ERK signaling pathway.

*DCN’s* function in the ovary is yet unknown, but its presence in the pre- and post ovulatory follicles and its induction by hCG suggest an important role in follicular development.

Based on previous studies, we explored two potential roles for *DCN*: An involvement in proliferation inhibition that was previously shown to be mediated by EGFR’, and a potential role in cell migration, as was also previously described for *DCN* in other cell types.

Our results show an increase in mRNA *DCN* levels in high-density cell cultures of mGCS in a dose-dependent pattern (Fig. 3a). However, adding exogenous DCN did not affect cell proliferation in MGC or KGN cell culture (Additional file 1). We presume that this model system may not be optimal for proliferation assays with *DCN*. Therefore, we believe that a role of *DCN* in the regulation of cell proliferation cannot be ruled out.

In addition to its possible role discussed above, *DCN* is described as a cell migration regulator [28, 33, 36], and mainly as an inhibitor. After the LH surge, GCs cells tend to migrate as part of a matrix formation process and as a prerequisite for ovulation and fertilization. We showed that *DCN* has an inhibitory effect on KGN cell migration (Fig. 4b). It has been recently shown that follicular *DCN* of oocytes fertilized by ICSI was significantly lower than that of the oocytes that were not fertilized [13]. This data together suggests that DCN may have a role in the regulation of MGCs’ migration, and may be responsible for attenuating migration during cumulus expansion and corpus luteum formation. Additional experiments are required in order to verify this theory.

Progesterone plays a major role in the pathogenesis and treatment of endometriosis. Progesterone resistance is a characteristic of the endometriotic tissue, probably due to downregulation of progesterone receptor (PR) in the ectopic tissue [51]. A previous study [37] suggested that induction of DCN by progesterone plays a crucial role in suppressing endometriosis. This phenomenon has been shown in endometrial cell lines [37]. Yoshihiro et al. showed that progesterone treatment promoted the expression and secretion of DCN in the immortalized human epithelial cells derived from an ovarian endometrioma. This study inspired us to examine this phenomenon on human GCs. Our data supports these findings: we demonstrated that MGC cultures treated with progesterone express higher levels of *DCN* compared to controls (Fig. 4a). Yoshihiro et al. suggested that progesterone anti-proliferative effects on immortalized human epithelial cells derived from an ovarian endometrioma are mediated by *DCN*. It was unclear whether *DCN* may be involved also in the pathophysiology of endometriosis. Interestingly, MGCs from women with endometriosis have abnormally low mRNA levels of *DCN* (Fig. 4b). Taken together, these results indicate that *DCN* expression in granulosa cells in the ovary might be involved in endometriosis, and further investigation of their role in this disease and the therapeutic role of progesterone may have clinical value.

In conclusion, our findings indicate that *DCN* is a novel ovulatory gene.

All in all, our results provide new insights into mechanisms that regulate the activity of *DCN* within the preovulatory follicles. A better understanding of *DCN*’s role in oocyte maturation and its involvement in ovulation and endometriosis may provide treatment for some types of infertility.

## Supplementary information

**Additional file 1.** Exogenous DCN does not inhibit MGCs or KGN proliferation in vitro.
