# Supplementary material for: Elucidating Decorin’s role in the preovulatory follicle
Source: J Ovarian Res. 2020 Feb 10;13:15. doi: 10.1186/s13048-020-0612-3 (PMC7011259; doi:10.1186/s13048-020-0612-3)
Supplement: Supplementary file 1 — Additional file 1. Exogenous DCN does not inhibit MGCs or KGN proliferation in vitro. [file 13048_2020_612_MOESM1_ESM.docx]

Supplement:

**Exogenous DCN does not inhibit MGCs or KGN proliferation *in vitro***

| High confluence areas | Low confluence areas |  |
| --- | --- | --- |
| 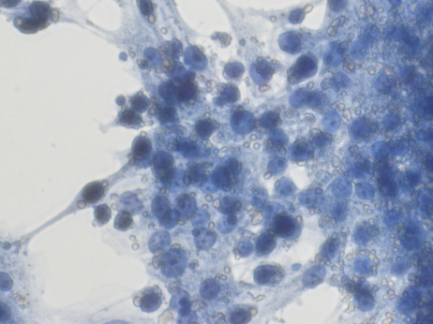 | 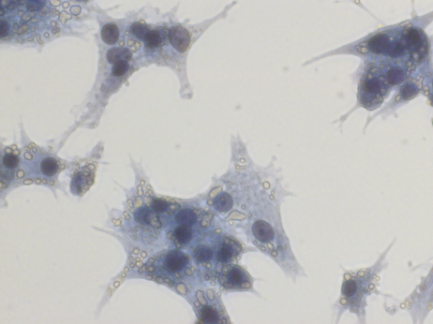 | Cont |
| 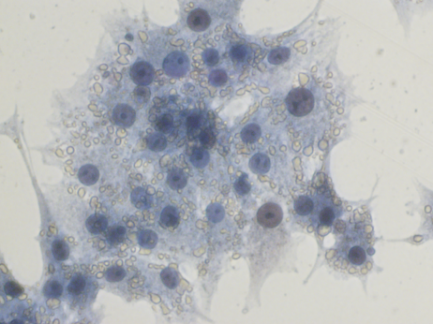 | 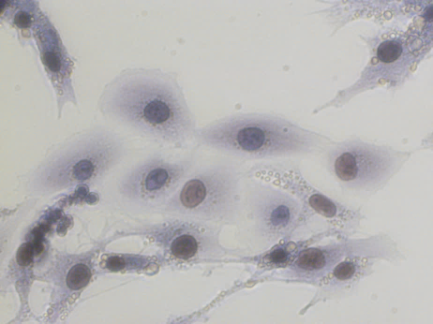 | 5µg/ml DCN |
| 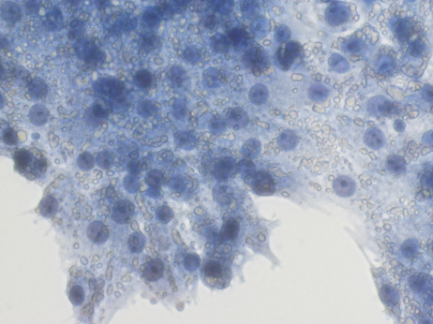 | 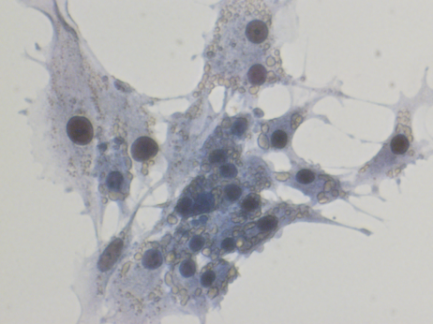 | 10µg/ml DCN |
| 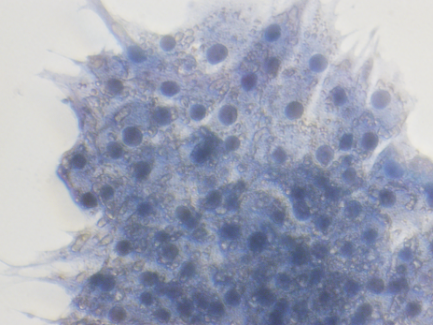 | 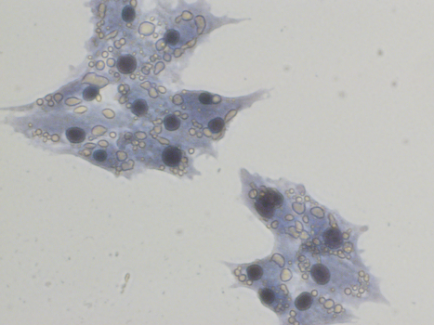 | 15µg/ml DCN |
| 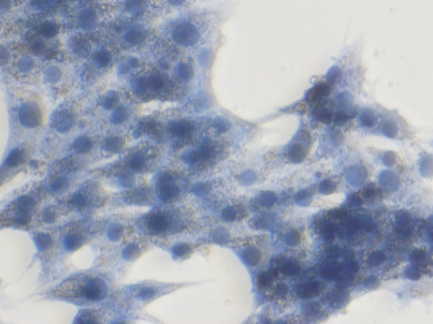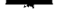 | 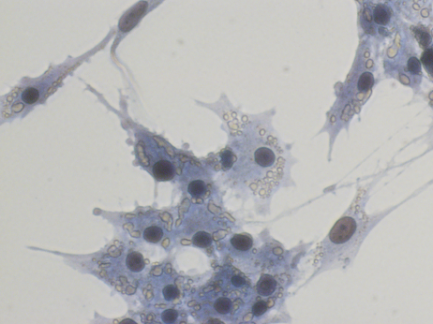 | 20µg/ml DCN |

Figure A: Immunohistochemical staining of PCNA in MGCs.
Cells were collected and cultured for 72h. Various concentrations of DCN as indicated were added daily. Cells were fixed and stained for PCNA. In each group two cell populations were exmamined, cells with low confluence and cells with high confluence. No noticble change in cell proliferation was obsurved in all concentrations compared to untreated control cells (X400) Bar=50µm.

| High confluence areas | Low confluence areas |  |
| --- | --- | --- |
| 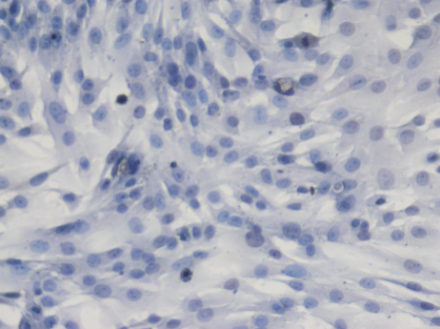 | 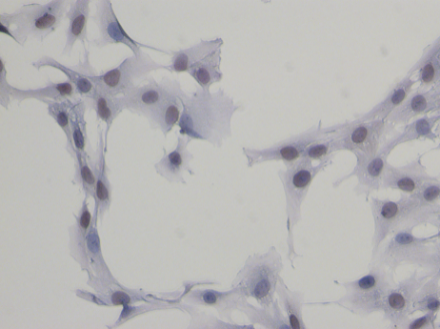 | Cont |
| 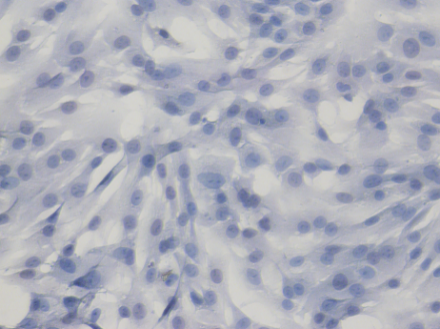 | 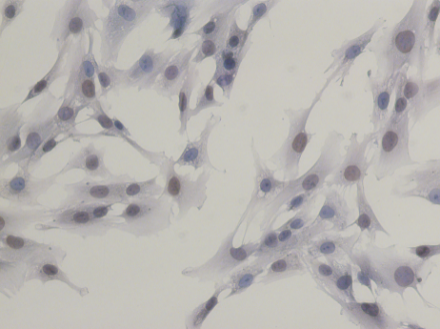 | 5µg/ml DCN |
| 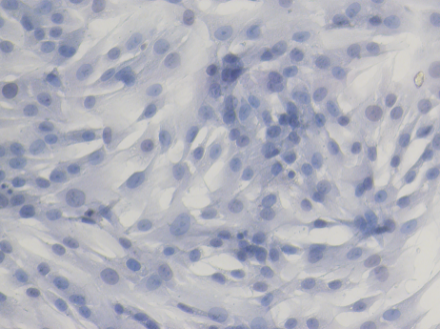 | 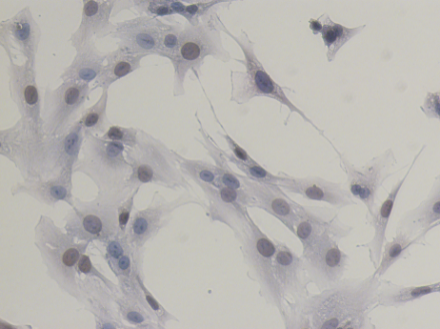 | 10µg/ml DCN |
| 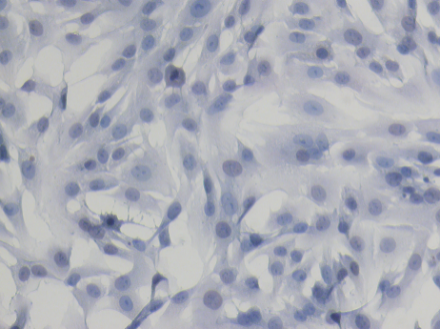 | 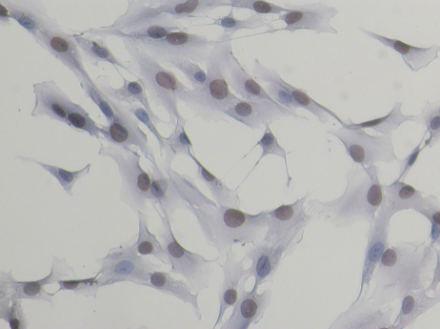 | 15µg/ml DCN |
| 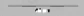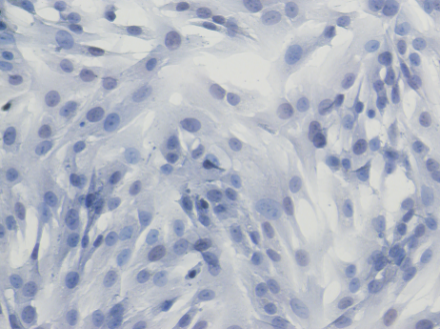 | 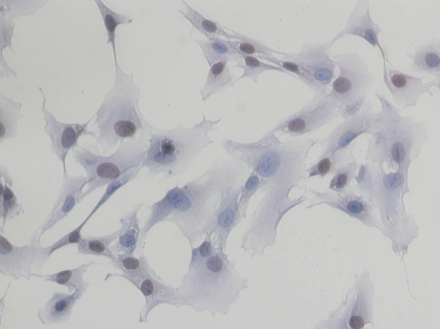 | 20µg/ml DCN |

**Figure B. Immunohistochemical staining of PCNA in KGN cells.** Cells were collected and cultured for 24h. Various concentrations of DCN were added daily. Cells were fixed and stained for PCNA. In each group two cell populations were exmamined, cells with low confluence and cells with high confluence. No noticble change in cell proliferation was observed in all concentrations compared to untreated control cells (X400) Bar=50µm.

**Exogenous decorin does not inhibit MGCs or KGN proliferation *in vitro***

Based on results exhibited in Fig. 3. We wanted to determine the involvement of DCN in MGCs and KGN proliferation regulation. We chose to examine the effect of DCN on MGCs and KGN proliferation using the PCNA method. (see M&M). MGCs were collected from women undergoing IVF and cultured. Medium was changed daily and various concentrations of DCN were added. Cells were cultured for 72h, fixed and stained for PCNA proliferation assay. KGN cells were cultured for 24h and treated the same way. In each sample two areas were examined, a dense high confluence area, and, a low confluence area with individual cells. Both DCN treated cells and control groups showed a similar proliferation pattern in which low confluence cells underwent proliferation unlike cells in denser areas that did not proliferate, probably due to contact inhibition. Both DCN treated cells and control groups showed a similar proliferation pattern in all DCN concentrations tested (Fig. A and Fig. B). Both, KGN and MGCs results support the notion that DCN does not inhibit cell proliferation *in vitro.*
